# Late-onset X-linked adrenal hypoplasia (DAX-1, *NR0B1*): two new adult-onset cases from a single center

**DOI:** 10.1007/s11102-017-0822-x

**Published:** 2017-07-24

**Authors:** Nikolaos Kyriakakis, Tolulope Shonibare, Julie Kyaw-Tun, Julie Lynch, Carlos F. Lagos, John C. Achermann, Robert D. Murray

**Affiliations:** 1 Department of Endocrinology, Leeds Centre for Diabetes & Endocrinology, St James’s University Hospital, Leeds Teaching Hospitals NHS Trust, Beckett Street, Leeds, LS9 7TF UK; 2 Leeds Institute of Cardiovascular and Metabolic Medicine, University of Leeds, Leeds, UK; 3 Department of Endocrinology, School of Medicine, Pontificia Universidad Católica de Chile, Santiago, Chile; 4 Facultad de Ciencia, Universidad San Sebastián, Campus Los Leones, Lota 2465 Providencia, 7510157 Santiago, Chile; 5 Genetics & Genomic Medicine, UCL Great Ormond Street Institute of Child Health, London, UK

**Keywords:** DAX-1, Adrenal hypoplasia congenita, Adrenal insufficiency, Hypogonadotropic hypogonadism, Repression helix domain, Nuclear receptors

## Abstract

**Purpose:**

DAX-1 (*NR0B1*) is an orphan nuclear receptor, which plays a critical role in development and regulation of the adrenal gland and hypothalamo–pituitary–gonadal axis. Mutations in *NR0B1* lead to adrenal hypoplasia congenita (AHC), hypogonadotropic hypogonadism (HH) and azoospermia in men. Presentation is typically with adrenal insufficiency (AI) during infancy or childhood. To date only eight cases/kindreds are reported to have presented in adulthood.

**Methods:**

We describe two new cases of men with DAX-1 mutations who presented in adulthood and who were diagnosed at a large University Hospital.

**Results:**

Case 1 presented with AI at 19 years. At 38 years he was diagnosed with HH. Detailed history revealed a brother diagnosed with AI at a similar age. Sequencing of the DAX-1 (*NR0B1*) gene revealed a heterozygous c.775T > C substitution in exon 1, which changes codon 259 from serine to proline (p.Ser259Pro). Case 2 was diagnosed with AI at 30 years. Aged 37 years he presented with HH and azoospermia. He was treated with gonadotropin therapy but remained azoospermic. Testicular biopsy showed maturational arrest and hypospermatogenesis. Analysis of the *NR0B1* gene showed a heterozygous c.836C > T substitution in exon 1, resulting in a change of codon 279 from proline to leucine (p.Pro279Leu). This change alters the structure of the repression helix domain of DAX-1 and affects protein complex interactions with NR5A family members.

**Conclusions:**

We describe two missense mutations within the putative carboxyl-terminal ligand binding domain of DAX-1, presenting with AHC and HH in adulthood, from a single center. DAX-1 mutations may be more frequent in adults than previously recognized. We recommend testing for DAX-1 mutations in all adults with primary AI and HH or impaired fertility where the etiology is unclear.

## Introduction

Primary adrenal insufficiency (AI) is a potentially life-threatening condition that results from a number of differing etiologies including autoimmune, genetic, and developmental disorders. The etiology is important for establishing potential associated comorbidities, inheritance, and optimal management. DAX-1 (dosage sensitive sex reversal, adrenal hypoplasia, critical region on the X chromosome, gene 1) is an orphan nuclear receptor encoded by the *NR0B1* gene. DAX-1 has a characteristic carboxyl-terminal ligand-binding domain (LBD) and an atypical DNA binding domain. The gene is located on the short arm of the X chromosome, in the Xp21 region, and plays a critical role in the embryological development of multiple endocrine tissues. It is expressed in the hypothalamus, pituitary gland, adrenals and gonads. After completion of tissue development DAX-1 continues to play a role in the regulation of hormone production.

Clinically, mutations in DAX-1 result in the X-linked form of primary adrenal hypoplasia congenita (AHC), hypogonadotrophic hypogonadism (HH) and azoospermia in men [1, 2]. Classically, males present in infancy or early childhood, with primary adrenal failure or isolated mineralocorticoid deficiency, with HH becoming apparent in adolescence by absent or arrested pubertal development [3]. Females are carriers and therefore generally unaffected. A spectrum of clinical presentations have been described, including delayed onset of AI and partial HH or even transient early puberty [4, 5]. In contrast, the description of DAX-1 *(NR0B1)* gene mutations presenting with onset of AI and HH in adulthood are rare.

We present two men with adult-onset AI and HH, who were found to have DAX-1 mutations, in addition to reviewing the previously described cases/kindreds of DAX-1 mutations presenting during adulthood.

## Case 1

A 42-year-old man with known cystic fibrosis (CF) and secondary diabetes was referred to Endocrinology for management of his Addison’s disease and androgen replacement. He was diagnosed with CF aged 4 years. As a consequence of CF he suffered mild abrogation of growth, with a final height of −2.5 SDS. His corresponding weight was −1.4 SDS, and pubertal development was assessed to be Tanner stage 5 at age 16. At age 18 years he was diagnosed with CF-related diabetes mellitus requiring insulin. During admission with a respiratory exacerbation of his CF at age 19 years he was noted to be pigmented, with serum sodium 124 mmol/L and potassium 5.1 mmol/L. Further tests confirmed cortisol insufficiency [9am cortisol <50 nmol/L (<1.8 µg/dL)] in association with unrecordable aldosterone [<55pmol/L (<1.5 ng/dL)]. Plasma renin activity is not available from the time of diagnosis. The patient was diagnosed with Addison’s disease and commenced on hydrocortisone and fludrocortisone replacement. At the time of the AI diagnosis the patient was on a regular Budesonide inhaler. However he was not on any oral medications (i.e. oral steroids or imidazoles), which could have caused iatrogenic adrenal insufficiency.

**Fig. 1:**
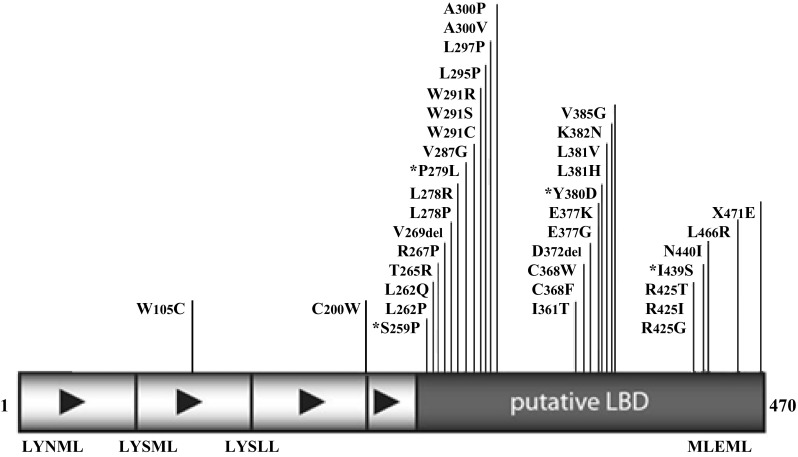
Schematic diagram of the DAX-1 protein showing reported missense mutations [6].* Asterisk* represent mutations associated with adult presentation of Adrenal Hypoplasia Congenita and Hypogonadotropic Hypogonadism (Modified with permission from Lin et al. Copyright © 2006 by The Endocrine Society)

At 35 years of age he underwent lung transplantation for end-stage pulmonary disease, necessitating long-term corticosteroid and immunosuppressive therapy with tacrolimus and mycophenolate mofetil. At age 38 years he presented with symptoms of low libido, was found to have HH [LH 1.6 IU/L, FSH 10.2 IU/L, testosterone 3.3 nmol/L (95 ng/dL), SHBG 48 nmol/L, estradiol 54pmol/L (14.7 pg/mL)], and was commenced on testosterone replacement. At the time of diagnosis of HH the patient was on hydrocortisone, as well as tacrolimus and mycophenolate mofetil. He was then lost to endocrine follow-up, however, on re-presentation aged 42 years, a detailed history revealed he had a brother diagnosed with Addison’s disease at a similar age. Investigations of additional pituitary axes were within normal limits [TSH 1.29 miu/L (reference range 0.2–4.0), free T4 17.9 pmol/L (reference range 10.0–20.0), prolactin 278 miu/L (normal level <600)], and a pituitary MRI was unremarkable. Neither the patient nor his brother have fathered children. Given the presence of Addison’s disease with HH, potential infertility, and family history, the patient was tested for mutation in the DAX-1 (*NR0B1*) gene. DNA analysis revealed a heterozygous c.775T > C (p.Ser259Pro) substitution within exon 1 of the DAX gene, which changes codon 259 from serine to proline in the putative LBD (Fig. 1). The results of in silico analysis suggested causality. p.Ser259 is a highly conserved amino acid in the ligand binding domain of the nuclear hormone receptor core of DAX1. SIFT (http://www.blocks.fhcrc.org/sift) predicts that p.Ser259Pro will affect protein function. Polyphen (http://www.genetics.bwh.harvard.edu) predicts p.Ser259Pro to be probably damaging. Russell analysis (http://www.russell.embl.de/ass) predicts the amino acid substitution from serine to proline to be disfavoured. Splicing (Alamut) c.775T > C is predicted that the variant does not affect the splicing of DAX1. The patient has declined genetic counseling, as he has not had any family plans so far.

## Case 2

A 30-year-old man presented with hyponatremia and hyperpigmentation. The presence of AI was confirmed with a short synacthen test (stimulation with 250 micrograms of synacthen), which revealed a baseline cortisol of 94 nmol/L (3.4 µg/dL) and a peak cortisol of 112 nmol/L (4 µg/dL). Serum aldosterone was <55 pmol/L (<1.5 ng/dL); however plasma renin activity is not available from the time of diagnosis. Adrenal antibodies were negative. He was diagnosed with Addison’s disease and commenced on hydrocortisone and fludrocortisone. At that time it was commented that his secondary sexual characteristics were normal, however, his testes were small. Gonadotropins showed LH 4.0 IU/L and FSH 10.0 IU/L, however a testosterone from that time is not available.

**Fig. 2:**
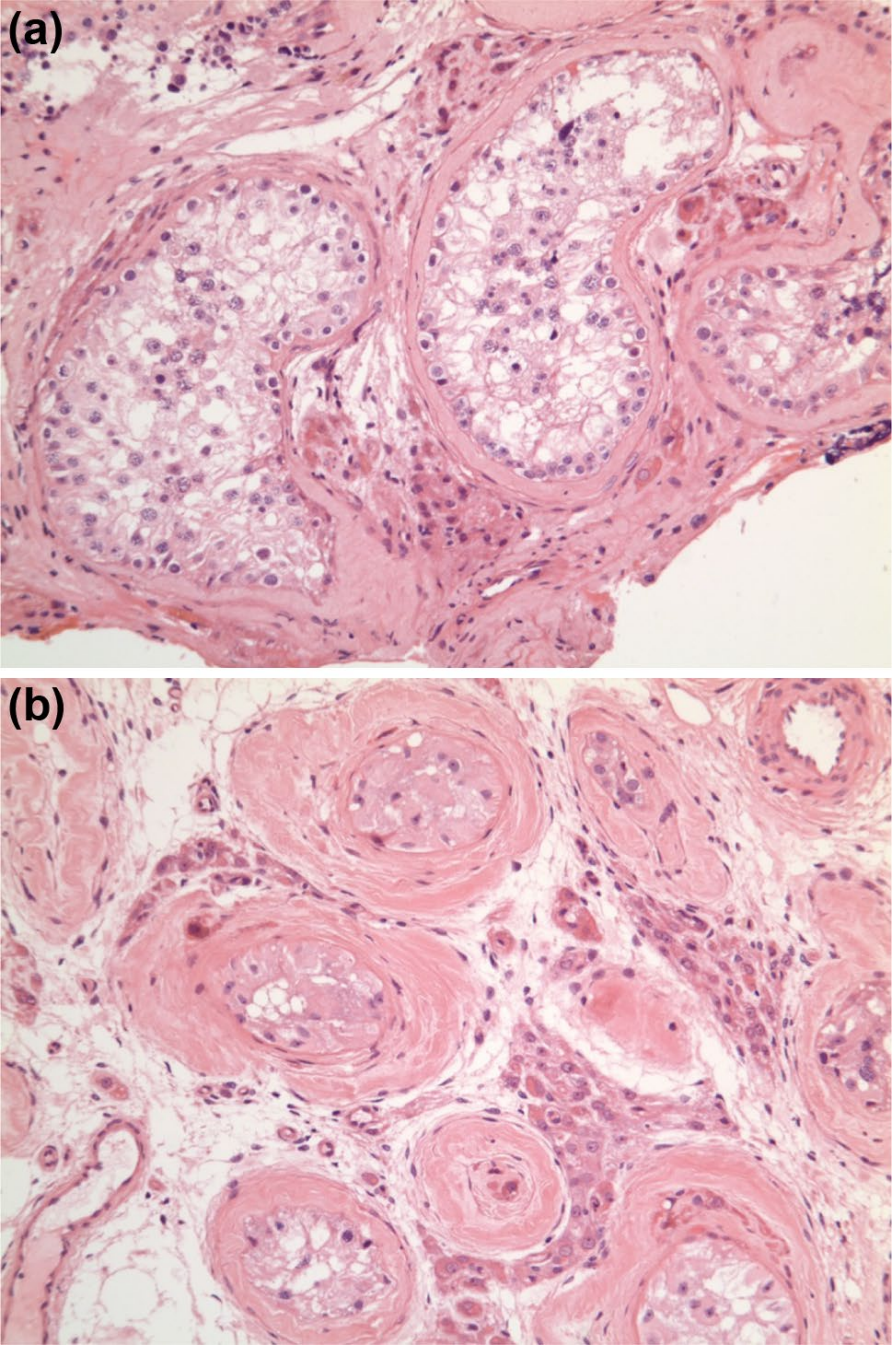
Histology obtained from testicular biopsy of patient with hemizygous c836C > T substitution in exon 1 of the DAX1 (NR0B1) gene. This missense mutation occurs within a ‘hot spot’ in the putative ligand binding domain which changes codon 279 from proline to leucine (p.Pro279Leu); **a** Demonstrates maturational arrest of spermatogenesis. The tubules contain germ cells but in low numbers. There is maturation up to primary spermatids but absent spermatazoa; **b** Demonstrates hyalinised and atrophic tubules (Sertoli cells only). Leydig cells are observed between the tubules. They are more easily seen compared to normal testis because of the general paucity of tubules (pseudohyperplasia)

**Fig. 3:**
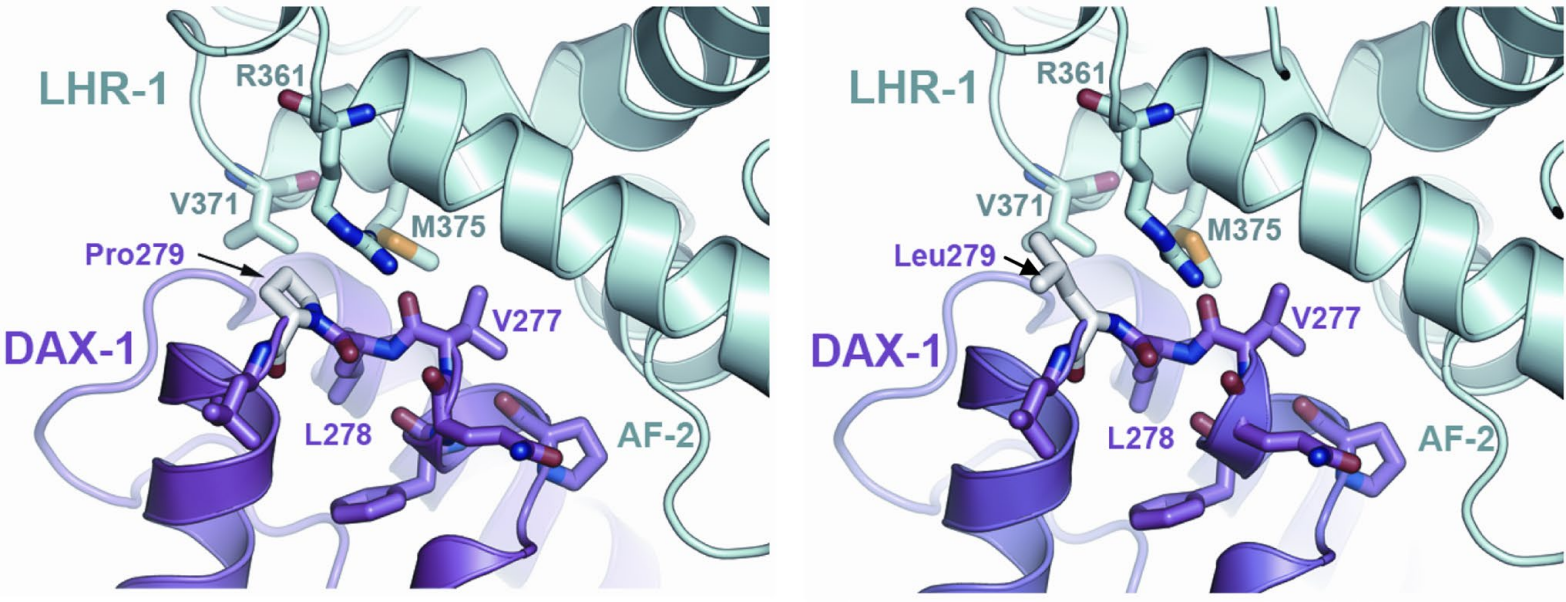
Model of the effect of the p.Pro279Leu mutation on the DAX-1:LRH-1 complex. The proline at 279 (shown in *white, left panel*) flanks the core repression helix domain of DAX-1 (*magenta*). The change to leucine (shown in *white, right panel*) likely increases the flexibility of the loop and produces a small displacement of an arginine side chain in LRH-1 (*cyan*). The comparative modelling of the human DAX-1: LHR-1 (Uniprot codes P51483 and O00482) complex was performed using the MODELLER program [7], as implemented in the Build Homology Model module of Discovery Studio v2.1 software (Accelrys Inc., San Diego, USA). The crystal structure of murine DAX-1:LHR-1 complex (PDBid 3F5C) solved at 3.0 Å resolution was used as template [8]. The protein complexes for the wild type and DAX-1 p.Pro279Leu mutant were modelled as (DAX-1)_2_:LHR-1 trimers, following the crystal structure oligomerization state. The resultant models were energy minimized using the conjugate gradient algorithm with the CHARMM22 force field until a RMS gradient of 0.001 kcal/molÅ was reached; a dielectric constant of 80 and a distance-dependent dielectric model was used during minimization [9]

Seven years later he presented to the Urology department with ejaculatory failure and subfertility. On examination his secondary sexual characteristics were documented to be consistent with androgen deficiency. He had a small left testis and an impalpable right testis due to a hydrocele. Semen analysis and post-ejaculatory urine showed azoospermia. His initial pituitary hormone profile revealed LH <0.5 IU/L, FSH 5.5 IU/L and testosterone <0.9 nmol/L (26 ng/dL). His remaining pituitary hormone profile was unremarkable [TSH 0.84 miu/L (reference range 0.2–4.0), free T4 13.8 pmol/L (reference range 10–20), random GH <0.1 mcg/L, IGF-1 36.9 nmol/L (reference range 10.1–28.4), prolactin 77 miu/L (normal level <600)], and pituitary imaging revealed no abnormalities. GnRH stimulatory test showed peak LH and FSH of 1.0 and 8.4 IU/L respectively. He was commenced on hCG and hMG injections which normalised his testosterone level [22.1 nmol/L (636 ng/dL)], sexual function, and secondary sexual characteristics, however, he remained azoospermic. He was referred to the Assisted Conception Unit and testicular sperm extraction (TESE) was attempted, but failed. Histological examination of his testis showed maturational arrest and hypospermatogenesis (Fig. 2). He was commenced on androgen replacement and continued his management under the Endocrine team. The association of AI and HH with poor response to fertility treatment prompted testing of the DAX-1 (*NR0B1*) gene. The test was performed by direct sequencing and revealed a hemizygous c836C > T substitution in exon 1. This missense mutation occurs within a ‘hot spot’ in the putative ligand binding domain which changes codon 279 from proline to leucine (p.Pro279Leu) (Fig. 1). The mutation has not previously been reported on the Human Genome Mutation Database and is not listed as a single nucleotide polymorphism (SNP) using the SNP-checker software. Investigation of the possible pathogenicity of this variation using Alamut software, as well as modelling, revealed that the sequence change occurs in both a highly conserved nucleotide and highly conserved amino acid that likely interacts directly with NR5A1/SF-1 (Fig. 3). The patient has not required genetic counseling with regards to his DAX-1 mutation, as previous investigations have shown infertility. He and his partner have now become parents to a healthy child following assisted conception via donor insemination.

Table 1 summarizes the endocrine investigations which led to the diagnosis of primary AI and HH in both our cases.

**Table 1:** Summary of the endocrine investigations performed in the two adult cases of DAX-1 (*NR0B1*) gene mutations (*n*/*a* not available)

Test	Case 1	Case 2	Reference range
9am Cortisol (nmol/L)	<50	Not performed	150–600
Short Synachten Test (nmol/L)	Not performed	94 (cortisol at 0 min) 112 (cortisol at 30 min)	Cortisol >500 nmol/L at 30 min
Aldosterone (pmol/L)	<55	<55	150–850
Plasma renin activity (nmol/L/h)	n/a	n/a	0.5–3.5
Testosterone (nmol/L)	3.3	<0.9	8.0–30.0
LH (IU/L)	1.6	<0.5	1.0–9.0
FSH (IU/L)	10.2	5.5	1.0–9.0
SHBG (nmol/L)	48	n/a	13–71
Prolactin (miu/L)	278	77	<600
TSH (miu/L)	1.2	0.84	0.2–4.0
Free T4 (pmol/L)	17.9	13.8	10–20
GH (mcg/L)	n/a	<0.1	–
IGF-1 (nmol/L)	n/a	36.9	10.1–28.4

## Discussion

We describe two unrelated men who demonstrate clinical presentation of X-linked adrenal hypoplasia during adulthood. In keeping with the majority of previous descriptions, both cases presented with AI prior to HH. In our patients AI was diagnosed at the age of 19 and 30 years, with hypogonadism diagnosed at 38 and 37 years respectively. Both DAX-1 mutations detected within our cases lie within the putative ligand binding domain (LBD).

The *NR0B1* gene encodes a 51 kDa protein which is part of the orphan nuclear receptor superfamily. The amino terminus containing three LXXLL-like motifs is implicated in protein–protein interactions, and the carboxyl terminus is a classic LBD. DAX-1 regulates development and function of the adrenal cortex and hypothalamo–pituitary–gonadal axis [1, 2], primarily by repression of gene expression. In part its actions are mediated by repression of another nuclear receptor, steroidogenic factor-1 (SF-1, *NR5A1*) [10]. Mutations in DAX-1/*NR0B1* classically present in males during infancy or childhood with primary adrenal failure or less commonly isolated mineralocorticoid deficiency, with hypogonadism becoming apparent as failure to enter or progress through puberty [3, 11]. A further feature of mutations in DAX-1 is a primary defect in spermatogenesis [12], characterized typically by failure to respond to exogenous gonadotropins [11, 13]. A single birth has been reported after finding rare spermatozoa in a man with HH resulting from a DAX-1 mutation after TESE-ICSI [14]. In addition to the more classic presentation, isolated HH in a female homozygous for mutations in *NR0B1* [15]; extreme pubertal delay in heterozygous female carriers [13]; 46,XY gonadal dysgenesis resulting from duplication of Xp21.2 [16]; and AHC as part of skewed X inactivation [17] have been described in females. More than 80 mutations in DAX-1 have been described, the majority of which are nonsense or frameshift mutations resulting in a truncated protein [5, 18]. Deletion of as few as the last nine amino acids of the DAX-1 protein, constituting the putative activation function-2 domain, is associated with a severe clinical phenotype [19]. Less common are missense mutations which predominantly cluster within the putative carboxyl LBD [11, 20]. Considerable variability in presentation, even within affected family members, is described, relating primarily to timing of presentation of adrenal insufficiency [1, 21, 22], although there is a suggestion that younger brothers are diagnosed earlier, likely representing increased awareness [23].

To date we are aware of only eight other reports of DAX-1 mutations in cases/kindreds presenting in adulthood (Table 2) [22, 24–29]. Three of these are associated with amino-terminal nonsense changes in DAX-1, and five are associated with carboxyl-terminal missense mutations. Table 2 summarizes the clinical cases of adult-onset adrenal insufficiency and hypogonadotrophic hypogonadism associated with mutations of the DAX1 (NR0B1) gene. Most of these patients presented with AI; however in two cases HH was the initial presentation, with the diagnosis of AI occurring at a later stage following further endocrine investigations. A recent case report described the same p.Ser259Pro mutation, as we found, in two Korean brothers who presented with primary AI at the age of 28 and 36 years respectively without evidence of hypogonadism [29]. The mutant protein showed reduced expression and impaired DAX-1 function in an in vitro assay. Clearly, these men will need follow-up, as hypogonadism may occur several years later, based on our experience.

**Table 2:** Summary of clinical cases of adult-onset adrenal insufficiency and hypogonadotrophic hypogonadism associated with mutations of the DAX-1 (*NR0B1*) gene

References	Age at presentation (years)	Presentation	Additional axis	Family history	Fertility	Genetic analysis
[28]	28	AI (↓peak cortisol to SST, ↑ACTH, ↓renin)	HH (↓testosterone, 6 ml testes, severe oligospermia)	Mother: heterozygous	No response to 10 months gonadotropin therapy	Missense p.I439S
[27]	28	HH (↓testosterone, 5 ml testes, azoospermia)	AI (↑ACTH, ↓peak cortisol to SST, normal renin)	Mother: heterozygous	No response to 8 months gonadotropin therapy	Missense p.Y380D
[24]	20	AI (↓cortisol, ↑ACTH, ↓renin)	HH (↓testosterone, 4 ml testis, ↓inhibin, azoospermia)	–	No response to 6 months gonadotropin therapy	Nonsense p.Q37X
[22]	18	AI (↓peak cortisol to SST, ↑ACTH, ↓renin)	HH (↓testosterone, azoo-spermia)	Brother (proband): AI age 5 years, HH age 21 years Mother: heterozygous	No response to gonadotropin therapy	p.Gln305Hisfs*67 (Deletion 305delG)
[26]	22	HH (delayed puberty, 2–3 ml testis, ↓testosterone)	AI (↓Na^+^, ↑K^+^, ↑ACTH, ↓peak cortisol to SST)	Brother: 18 years, HH, ↑ACTH, ↓peak cortisol to SST	–	Nonsense p.W39X
[25]	19	AI	Age 24 years: normal testes volumes and LH/FSH/inhibin B levels; oligospermia	Mother: heterozygous Sister: heterozygous Nephew: AI crisis age 2 weeks Brother: 36 years, ↓peak cortisol to SST, ↓testosterone, oligospermia	Successful IVF age 33 years. Spontaneous pregnancy age 35 years	Nonsense p.W39X
[29]	28	AI (↓cortisol, ↑ACTH, adrenal hypoplasia on CT imaging	No other axes affected (normal testosterone)	Brother: AI age 36 years, testosterone not done, genetic analysis—Missense p.S259P Mother: healthy, genetic analysis—Missense p.S259P Maternal cousin: primary AI, no genetic test performed	Normal	Missense p.S259P
Kyriakakis (2017) (Case 1)	19	AI (↓Na+,↑K+, ↓peak cortisol to SST)	Age 38 years: HH, ↓testosterone	Brother: AI, no children	No children	Missense p.S259P
Kyriakakis (2017) (Case 2)	30	AI (↓peak cortisol to SST)	Age 37 years: HH, small testis, azoospermia		No response to gonadotropin therapy. No mature spermatozoa on TESE	Missense p.P279L

*AI* adrenal insufficiency, *HH* hypogonadotrophic hypogonadism, *IVF* in vitro fertilization, *SST* short synacthen test, *TESE* testicular sperm extraction

To date, the described mutations within DAX-1 kindreds which have at least one case presenting in adult life include p.Q37X, p.W39X in two kindreds, p.S259P in three patients, p.P279L, p.Gln305Hisfs*67 (c.915delG), p.Y380D, and p.I439S (Table 2). The first two of these mutations result in a functional DAX-1 protein from an in-frame translational site downstream to the premature stop codon [24–26]. The next four of the described mutations are missense mutations widely distributed throughout the carboxyl LBD, but that may form important structural components of the protein structure or affect subcellular localisation [27, 28]. The final described mutation was a frameshift within the LBD [22].

Our patients add to previous reports of late-onset X-linked AHC presenting in adulthood, and expand the list of DAX-1 mutations that can present in this way. The p.Pro279Leu mutation is a novel missense mutation within the LBD, which to our knowledge has not been previously reported in the literature. Based on the crystal structure of DAX-1 bound to NR5A2/LRH-1, this codon is one of two highly conserved proline residues flanking a unique repression helix (RH) domain that interacts directly with NR5A2 (Fig. 3) [8]. The core of this RH domain is a variant “LXXLL” motif containing exposed hydrophobic residues (Human sequence: 273_279PCFQVLP; Mouse sequence: 275_281PCFQILP). This region of DAX-1 blocks the interaction of co-activator with NR5A2, resulting in transcriptional repression. The proline at 279 flanks the core region. The change to leucine increases the flexibility of the loop and produces a small displacement of an arginine side chain in LRH-1. This change causes partial disruption of the protein–protein interaction and a delayed-onset phenotype. Of note, p.Leu278Arg and p.Leu278Pro variants affecting the core leucine have been reported in patients with classic early-onset X-linked adrenal hypoplasia.

The p.Ser259Pro mutation, which is the most amino-terminal mutation described in the putative LBD to date (Fig. 1), has also been recently reported in two Korean brothers with late-onset primary AI and no evidence of hypogonadism [29]. The authors demonstrated that the mutation caused a loss of the NR0B1 gene function, as proven by the lower NR0B1 protein levels in cells with NR0B1 mutation (measured by western blot) compared with wild type cells [29]. The vast majority of missense mutations are reported to occur in the putative carboxyl terminal LBD, specifically within two clusters between codons 262–300 and 361–385, with a scattering of further missense mutations immediately adjacent to the carboxyl terminus [5]. Both the mutations we describe fall around the first of these “hotspots”.

Genetic analyses in our patients were performed by bi-directional sequencing of exons 1 and 2 of *NR0B1*. A limitation to our case reports is that the results of some endocrine investigations are not available for our patients, including the values of the ACTH and plasma renin activity at the time of diagnosis of primary AI. This is because in both cases, the initial diagnosis of primary AI was made approximately 20 years prior to the genetic confirmation of the mutation in the DAX-1 gene and therefore some historic medical data is no longer available in the patients’ hospital records.

Adult presentation of patients with DAX-1 mutations likely reflects partial, rather than complete loss of repressor activity, as demonstrated in transient gene expression studies and from structural predictions [24, 27, 28]. The finding of two unrelated cases within our center, where we follow-up approximately 40–50 cases of primary AI, suggests presentation of DAX-1 during adulthood may be under-recognized. This hypothesis was investigated in a cohort of 29 male and female patients with adult-onset AI where common causes including autoimmune disease, steroidogenic defects, and metabolic disorders (e.g. adrenoleukodystrophy) had been excluded. Within this cohort no patients were found to have a DAX-1 mutation [6]. However, none of these patients were reported to have concurrent hypogonadism. The current report strongly suggests that the combination of primary AI with HH at any age should raise the suspicion of DAX-1 gene mutation, and prompt genetic testing. Diagnosing X-linked AHC is clinically important for identifying siblings who could be at risk of life-threatening AI [26], identifying sisters who might have affected sons, and optimising approaches to endocrine replacement and fertility treatment. As the hypogonadism can occur some time after the diagnosis of adrenal insufficiency, awareness of this condition, careful monitoring, and possibly, genetic testing is warranted.

## Abbreviations

ACTH: Adrenocorticotropin hormone
AHC: Adrenal hypoplasia congenita
AI: Adrenal insufficiency
CF: Cystic fibrosis
DAX-1: Dosage sensitive sex reversal, adrenal hypoplasia, critical region on the X chromosome, gene 1
FSH: Follicle-stimulating hormone
hCG: Human chorionic gonadotropin
HH: Hypogonadotrophic hypogonadism
hMG: Human menopausal gonadotropin
GnRH: Gonadotropin releasing hormone
IU: International units
IVF: In-vitro fertilisation
kDA: Kilo Dalton
L: Litre
LBD: Ligand binding domain
LH: Luteinizing hormone
LRH: Liver receptor homolog-1
mmol: Millimole
nmol: Nanomole
NR0B1: Nuclear receptor subfamily 0, group B, member 1
NR5A1: Nuclear receptor subfamily 5, group A, member 1
NR5A2: Nuclear receptor subfamily 5, group A, member 2
pmol: Picomole
RH: Repression helix
SDS: Standard deviation scores
SF-1: Steroidogenic factor-1
SHBG: Steroid hormone binding globulin
TESE-ICSI: Testicular sperm extraction for intracytoplasmic sperm injection
